# Complete Cystic Degeneration of a Uterine Myoma Posing a Diagnostic Dilemma

**DOI:** 10.1155/crra/5627017

**Published:** 2025-05-01

**Authors:** Abdi Dandena Dibaba, Neema Ljani, Aqeela Mustafa, Hussein Khanbhai, Abel Ntungi, Alfred Secha, Natnael Alemu Bezabih, Misiker Gebremariam Waktola, Shafi Samiji Ramadhani

**Affiliations:** ^1^Department of Radiology, Faculty of Medical Science, Institute of Health, Jimma University, Jimma, Ethiopia; ^2^Dar es Salaam City Council, Dar es Salaam, Tanzania; ^3^Addis Ababa University, Addis Ababa, Ethiopia; ^4^Department of Pathology, Muhimbili University of Health and Allied Sciences, Dar es Salaam, Tanzania

**Keywords:** cystic degeneration, diagnostic dilemma, pelvic MRI, uterine myoma

## Abstract

In this case, we describe a rare presentation of a uterine myoma in a 42-year-old female patient who presented with a progressively enlarging abdominopelvic mass and heavy menstrual bleeding for 1 month. Initial work showed that the patient has a low hemoglobin count and elevated CA-125 tumor marker. A pelvic ultrasound revealed a cystic pelvic lesion with an undetermined origin. Subsequent follow-up after 6 weeks showed significant enlargement of the mass, prompting a pelvic MRI to be performed; the MRI showed a large, completely cystic uterine myoma arising from the anterior myometrium. This case highlights the atypical presentation of a common gynecological condition that can result in a diagnostic dilemma and the importance of advanced imaging such as MRI to be a problem-solving tool.

## 1. Introduction

Uterine fibroids also known as leiomyoma or myomas are the commonest uterine neoplasms [1]. The incidence and prevalence of uterine fibroids may vary widely, with some studies suggesting uterine fibroids are seen in approximately 70% of all women within the reproductive age group. One of the major risk factors that impact the incidence of fibroids is race. Black women have a two- to threefold increased risk of developing uterine fibroids compared to White women [2].

The exact pathogenesis of leiomyoma is not known; however, hormonal factors are incriminated due to the rarity of myomas before menarche and their regression postmenopause [3]. In addition, studies have shown evidence of factors that drive the development or exacerbation of myomas. Such evidences include lifestyle, age, environment, family history of uterine fibroids, and vitamin D deficiencies [4].

Uterine fibroids can have variable clinical presentation. It is estimated that 50% of fibroids are asymptomatic and are discovered incidentally during another test such as imaging or routine cervical screening [5]. The most frequent symptom is abnormal uterine bleeding which can at times lead to iron deficiency anemia and high output failure. Other symptoms include dyspareunia, abdominal pain, urinary complaints, constipation, and infertility [3].

## 2. Case Presentation

A 42-year-old female, para 3 with 2 living babies, attended her first gynecology visit on 10 June 2023, with complaints of a prolonged heavy menstrual period for more than 1 month which lasted for 8–10 days with heavy bleeding necessitating exchanging 4–6 pads per day, especially on the first 3–4 days; bleeding was fresh and clotted blood. On physical examination of the abdomen, a palpable abdominal swelling of the equivalent of 20/40 fundal height was noted.

Laboratory investigations were done, with her blood count showing anemia with hemoglobin of 5.5 g/dL. Additional tumor markers revealed elevated CA-125 (62.59 U/mL) and with normal levels of CEA (0.9 ng/mL) and AFP (4.15 IU/mL). Hormonal analysis was not done.

**Figure 1:**
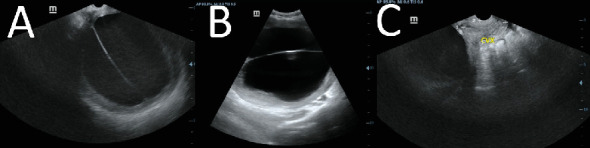
Initial ultrasound examination of the pelvis on the sagittal (A) and axial (B) images showed a large, well-defined cystic mass lesion in the pelvis with internal septations. The uterus and adnexa are not well visualized on scanning with only the laterally displaced cervix was visualized (C).

On pelvic ultrasound examination, a large complex cystic pelvic mass lesion was seen with thick internal septations. The cyst measured 17.10 × 15.28 × 20 cm in size and showed peripheral flow on color Doppler ultrasound interrogation; the internal septations did not show any color flow. The ovaries were not separately visualized during the scan, with an initial impression of a cystic pelvic mass of undetermined origin; therefore, further imaging by pelvic MRI was requested (Figure 1).

**Figure 2:**
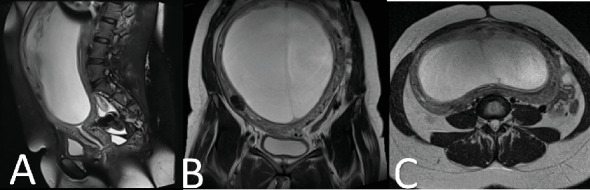
Multiplanar follow-up MRI on the pelvis in the (A) sagittal, (B) coronal, and (C) axial showing a large well-defined T2 hyperintense and T1 hypointense mass in the anterior wall of the uterine cavity, posteriorly effacing the endometrial cavity. There are additional multiple small different-sized T2 hypointense masses located within the myometrium of the uterus.

The follow-up visit after 6 weeks revealed a significant increase in the abdominal swelling to the equivalent of 30/40 fundal height from the prior 20/40 measurement. The MRI showed a large well-defined T2 hyper and T1 hypointense mass in the anterior wall of the uterine cavity measuring 23 cm (L) × 21 cm (W) × 11 cm (AP) in size, posteriorly effacing the endometrial cavity. There were additional multiple small different-sized T2 hypointense masses located within the myometrium of the uterus. The junctional zone appears normal in size measuring 2 mm in depth. The cervix measures 5 cm in length and has normal endocervical fluid signals, and no stenosis of internal OS was seen. No lesion was seen. The parametrium is normal. The ovaries were visualized separately and have normal size and signal (Figure 2).

**Figure 3:**
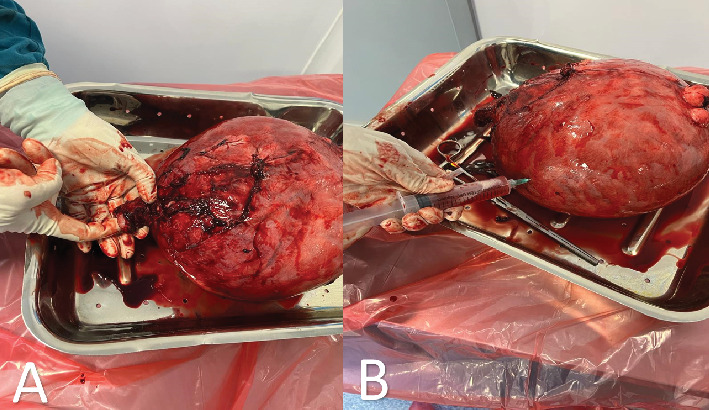
(A) Total hysterectomy specimen grossly weighed 1720 g, total dimensions: 20 × 12 × 8 cm. The specimen seem to be asymmetrically enlarged, and some subserosal pedunculated fibroids can be seen (B). Huge intramural fibroids with cystic generation (seen as serosanguinous fluid in the syringe) can be appreciated.

The patient underwent exploratory laparotomy, and a total abdominal hysterectomy was performed; the intraoperative findings showed a huge uterine myoma with complete cystic changes and additional multiple small fibroids about around the fundus of the uterus. Both ovaries were normal; the left ovary adhered to the uterus, a standard trans-abdominal hysterectomy (TAH) was performed, and the specimen was taken for histology (Figure 3).

The removed tumor measured dimensions of 20 × 12 × 8 cm and weighed 1720 g. Cut section: The endometrium is 0.5 cm thick and is polypoid. The myometrium has a thickness of 3 cm and is trabeculated. Fibroids: Number, multiple; location, intramural and submucosal; size, 1.5–12 cm; necrosis/hemorrhage, not identified. Cystic degeneration and yellowish areas are noted in the largest fibroid. The cut section shows a homogenous white whorled appearance. Sections from small fibroids. Representative sections are taken (13 blocks).

**Figure 4:**
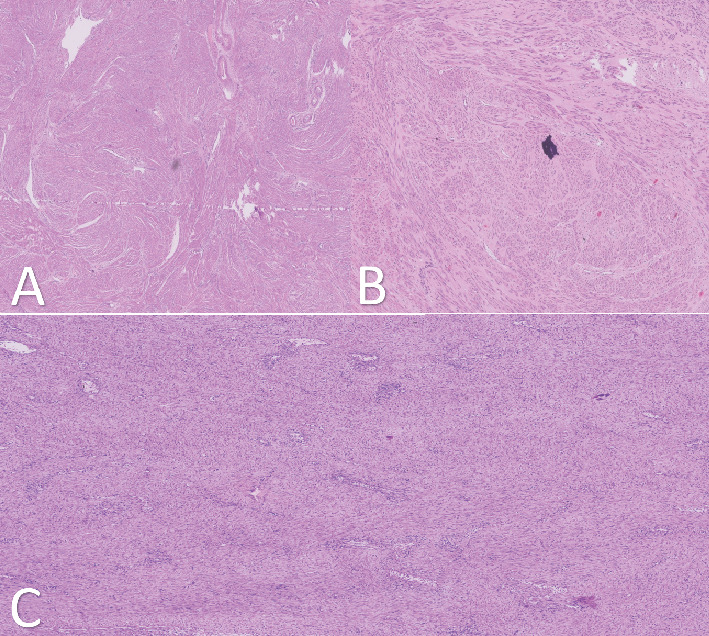
Sections of tissue show a tumor composed of interlacing fascicles of benign looking spindle-shaped cells within an edematous and hyalinized stroma. Secondary changes are also evident, that is, cystic degeneration (A) and dystrophic calcification (B) (hematoxylin and eosin stain ×100 magnification). (C) Stromal hyalinization and increased vascularity.

Microscopy endometrium: Suboptimal preservation there is no overt evidence of malignancy. Myometrium: Leiomyoma's multiple sections examined show a tumor composed of interlacing fascicles of benign spindle-shaped cells within an edematous and hyalinized stroma. No nuclear atypia or increased mitotic activity is noted. The largest fibroid is submucosal in location and shows foci of cystic degeneration and infarction change (Figure 4).

## 3. Discussion

Uterine fibroid is among the most common gynecological diseases seen in women in their reproductive age, and their diagnosis is often suspected from clinical history and physical examination and subsequently confirmed using imaging modalities. Often, the initial imaging modality for diagnosing fibroids is ultrasound, due to its accessibility and low cost. On ultrasound scanning, fibroids will typically appear as a well-defined solid mass lesion with a whorl appearance. They often tend to distort the uterine contour or make the uterus appear bulky. Typical fibroids tend to have a hypoechoic appearance with posterior acoustic shadowing. An acoustic showing pattern known as a “Venetian blind artifact” is a very typical sonographic feature of fibroids [6]. Doppler ultrasound can often show a typical peripheral flow pattern. Degenerated fibroids can have atypical sonographic features with heterogeneous echo texture often giving areas of calcification, and cystic changes are quite common. However complete cystic degeneration is very rare [7].

MRI is the imaging modality of choice in the evaluation of fibroids due to its supreme soft tissue contrast resolution and has shown to be superior compared to ultrasound in the characterization of fibroids. Some of the additional benefits of MRI in the evaluation of fibroids include the diagnosis of coexisting gynecological conditions that affect management decision such as adenomyosis and endometriosis. This is especially important for patients who are planning to undergo uterine salvage therapy [8]. Additionally, MRI has the added advantage of picking up fibroids in atypical locations such as the cervix and can also be useful in ambiguous cases where a pedunculated myoma and an adnexal mass cannot be discriminated on ultrasound alone such as seen in our case [9]. Typically, myomas appear as well-defined T2 hypointense lesions with intermediate to high signals on T1-weighted images connected to the uterus [8].

Although the clinical and imaging appearance of uterine myomas are well known, they can sometimes cause diagnostic dilemmas due to atypical presentations and/or atypical imaging appearance. Atypical appearances are generally attributed to the degeneration of the myomas which often result from an enlarging myoma that outgrows its blood supply leading to a cascade of inflammatory and ischemic changes finally ending in various types of degenerations including hyaline, myxoid, cystic, red, and calcific degenerations [10].

Myomas that undergo red degeneration will have an appearance of T1 hyperintense peripheral rim due to the T1 shortening effect of methemoglobin, while myxoid degeneration will appear to have a high signal intensity in the T2-weighted images showing a minimal progressive enhancement on postcontrast images [11].

Cystic degeneration is seen in 4% of uterine myomas. Usually, cystic degeneration occurs due to a rapidly growing myoma which will surpass the blood supply leading to degeneration. Cystic degeneration is considered to be an extreme sequel of edema [1]. Fully developed cystic degeneration is relatively rare and can present a diagnostic dilemma when distinguishing it from other frequently encountered pelvic cystic lesions, particularly in cases where the lesions are sizable, as frequently observed in prior reported instances of cystic degeneration of uterine myoma [12].

Moreover, the elevation of CA-125, a commonly assessed tumor marker for ovarian cancer, further compounded the confusion in this case, with the values nearly doubling the upper limits of normal expected ranges. However, it has been shown that the peripheral CA-125 levels can be increased in patients' uterine myomas, especially in larger myomas which can cause distention and irritation of the overlying peritoneum [13].

## 4. Conclusion

While most uterine myomas typically present with a distinctive clinical and ultrasound appearance, facilitating a straightforward diagnosis in the majority of cases, degenerative alterations in myomas can create diagnostic hurdles. In this patient, the rapid enlargement of the mass heightened CA-125 levels and the entirely cystic nature of the mass with prominent internal septations observed on ultrasound all contributed to diagnostic complexities. However, understanding the various imaging characteristics of uterine myomas and the utilization of advanced imaging techniques such as MRI can be crucial for confirming the diagnosis.

## Data Availability

The data that support the findings of this case report are available from Saifee Hospital Tanzania upon reasonable request. Restrictions apply to the availability of these data, which were used under license for this case report, and so are not publicly available. Data are however available from the authors upon reasonable request and with permission of Saifee Hospital Tanzania.

## Nomenclature

AFP: alpha-fetoprotein
CA: cancer antigen
CEA: carcinoembryonic antigen
TAH: total abdominal hysterectomy
UAE: uterine artery embolization
